# Pathogeny of cerebral venous thrombosis in SARS-Cov-2 infection: Case reports

**DOI:** 10.1097/MD.0000000000024708

**Published:** 2021-03-12

**Authors:** Cécile Guendouz, Véronique Quenardelle, Nolwenn Riou-Comte, Pascal Welfringer, Valérie Wolff, Stéphane Zuily, Lavinia Jager, Lisa Humbertjean Selton, Gioia Mione, Raoul Pop, Benjamin Gory, Sébastien Richard

**Affiliations:** a Department of Neurology, Université de Lorraine, CHRU-Nancy, Stroke Unit, CIC-P 1433, INSERMU1116, Nancy; b Stroke Unit, Strasbourg University Hospital, EA3072, Strasbourg; c Department of Anesthesia and Resuscitation, CHRU-Nancy; d Université de Lorraine, Inserm, DCAC and CHRU-Nancy, Vascular Medicine Division and Regional Competence Center for Rare Auto-Immune Diseases, Nancy; e Hôpital Marie-Madeleine de Forbach, Forbach; f Department of Neurology, CHRU-Nancy, Stroke Unit, Nancy; g Department of Interventional Neuroradiology, University Hospital of Strasbourg, Strasbourg; h Department of Diagnostic and Therapeutic Neuroradiology, Université de Lorraine, CHRU-Nancy, INSERM U1254, IADI, Nancy, France.

**Keywords:** cerebral infarction, cerebral venous thrombosis, COVID-19, SARS-Cov-2, stroke

## Abstract

**Rationale:**

Pathogeny of thrombosis in COVID-19 is related to interaction of SARS-Cov-2 with vascular wall through the angiotensin converting enzyme 2 (ACE2) receptor. This induces 2 pathways with immunothrombosis from activated endothelium (cytokine storm, leukocyte and platelet recruitment, and activation of coagulation extrinsic pathway), and rise of angiotensin II levels promoting inflammation. While thrombosis is widely described in COVID-19 patients admitted in intensive care unit, cerebrovascular diseases remains rare, in particular cerebral venous thrombosis (CVT).

**Patient concerns:**

We describe 2 cases of women admitted during the spring of 2020 for intracranial hypertension signs, in stroke units in Great-east, a French area particularly affected by COVID-19 pandemia.

**Diagnoses:**

Cerebral imaging revealed extended CVT in both cases. The first case described was more serious due to right supratentorial venous infarction with hemorrhagic transformation leading to herniation. Both patients presented typical pneumonia due to SARS-Cov-2 infection, confirmed by reverse transcription polymerase chain reaction on a nasopharyngeal swab in only one.

**Interventions:**

The first patient had to undergo decompressive craniectomy, and both patients were treated with anticoagulation therapy.

**Outcomes:**

Favorable outcome was observed for 1 patient. Persistent coma, due to bi thalamic infarction, remained for the other with more serious presentation.

**Lessons:**

CVT, as a serious complication of COVID-19, has to be searched in all patients with intracranial hypertension syndrome. Data about anticoagulation therapy to prevent such serious thrombosis in SARS-Cov-2 infection are lacking, in particular in patients with mild and moderate COVID-19.

## 1 Introduction

Thrombosis is observed in up to 70% of COVID-19 patients,^[1–5]^ despite thromboprophylaxis,^[1]^ and more than in any other severe acute respiratory syndrome (SARS).^[4]^ Venous thromboembolism (VTE) is the most observed, but some life-threatening arterial thromboses,^[6]^ as mesenteric,^[7]^ and leg vascular graft,^[8]^ are also described. In contrast, stroke is barely observed in studies, and cerebral venous thrombosis (CVT) reports remain scarce.^[9–23]^ More, all studies about thrombosis incidence, and reports of CVT cases, included COVID-19 patients admitted in intensive care unit (ICU), making it difficult to establish causality due to many other pro thrombotic factors.

We report 2 cases of patients with CVT in the context of COVID-19. Diagnosis of CVT was made as early as admission in emergency department. We discuss about pathogeny of thrombosis in COVID-19, and reasons for low reported incidence of cerebrovascular diseases.

## 2 Case summary

Next of kin for the first patient, and second patient gave written consent for collection of data from their medical report for scientific paper. All data have been anonymized.

## 3 Case 1

**Figure 1:**
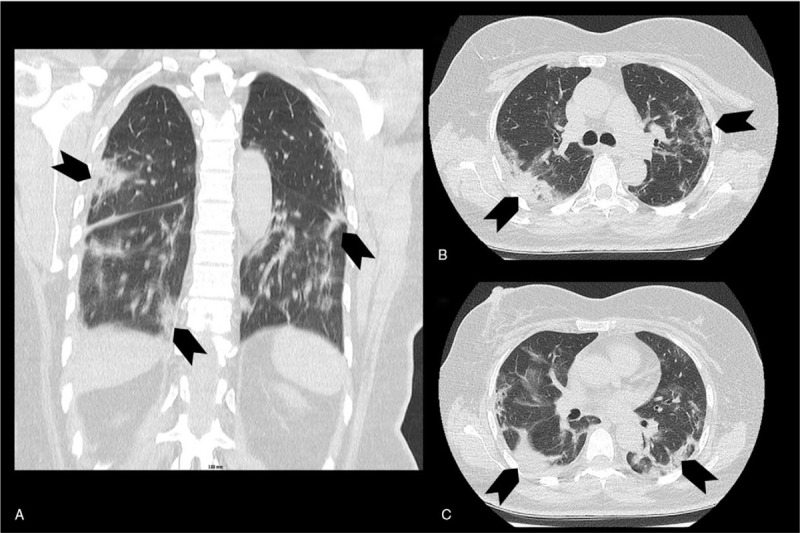
Patient (case 1) chest CT scan. Chest CT scan (A: frontal view, B and C: axial views) showing typical pneumonia due to SARS-Cov-2 infection with ground glass opacities (arrows).

**Figure 2:**
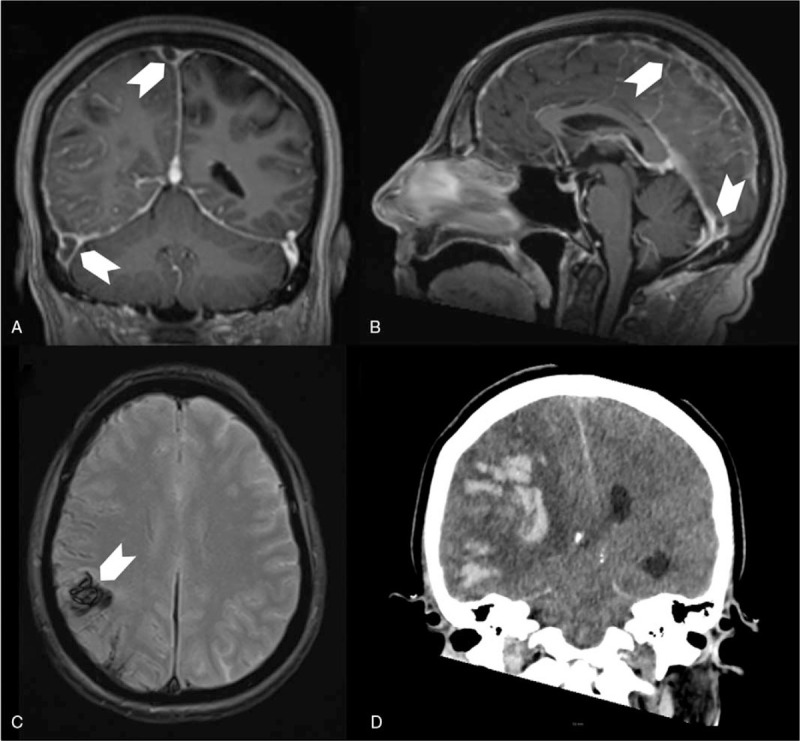
Patient (case 1) cerebral imaging showing cerebral venous thrombosis and venous infarction with hemorrhagic transformation. A (frontal view), and B (sagittal view): cerebral MRI T1 sequence with gadolinium showing filling defect in the superior sagittal sinus and the right lateral sinus (arrows), C (axial view): T2∗-weighted gradient-recalled echo resonance imaging showing right parietal hemorrhage (arrow), D (frontal view): 24-hour control cerebral CT scan showing extended venous infarction with hemorrhagic transformation leading to subfalcine and transtentorial herniation.

A 56-year-old woman with medical history of type 2 diabetes, hypothyroidism, and right breast cancer treated by surgery, radiotherapy, and antiestrogen, in remission since 5 years, was admitted at the Forbach Hospital (Great East, France) on March 30, 2020 at the pick of COVID-19 pandemia at this time in this area. The patient presented left hemiplegia and fever. While nasopharynx reverse transcription polymerase chain reaction (PCR) was negative for SARS-CoV-2, diagnosis of COVID-19 was made through chest CT scan revealing typical COVID-19 pneumonia with ground-glass opacities (Fig. 1). Cerebral MRI revealed thrombosis of the superior sagittal sinus, and the right lateral sinus spreading to the right internal jugular vein, with right subarachnoid hemorrhage, and parietal intraparenchymal hematoma (Fig. 2). Cerebrospinal fluid was hemorrhagic with 3 leukocytes per μl, and high level of protein (225 mg/dl). Blood tests showed hemoglobin level of 12 g/dl, platelet count at 320 G/L, increased leukocyte count at 12 G/L, with neutrophil count at 11G/L, and decreased lymphocyte count at 0.8 G/L. C-reactive protein level was increased at 54 mg/L. Coagulation tests showed normal prothrombin time (PT), activated partial thromboplastin time (APTT), protein C, and protein S levels, no activated protein C resistance, and no antiphospholipid antibodies (including antiβ2GP1 antibodies), but slight decrease of antithrombin activity (73%). D-Dimer level was not assessed. Anticoagulant therapy with unfractionated heparin was started after admission in stroke unit. On March 31, 2020, clinical worsening was observed with coma and right mydriasis requiring admission in ICU. Control cerebral CT scan showed extended venous infarction with hemorrhagic transformation leading to subfalcine and transtentorial herniation (Fig. 2). Despite urgent decompressive craniectomy performed in the University Hospital of Nancy (Great East, France) and anticoagulant therapy, outcome was poor with bi thalamic infarction and persistent coma.

## 4 Case 2

**Figure 3:**
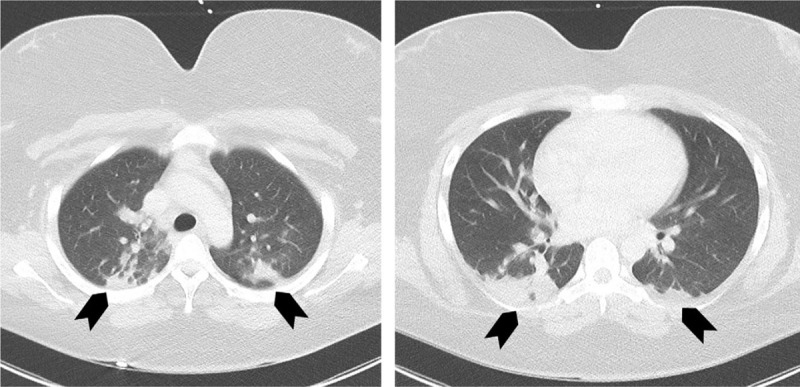
Patient (case 2) chest CT scan. Chest CT scan (axial views) showing typical pneumomia due to SARS-Cov-2 infection with ground glass opacities (arrows).

**Figure 4:**
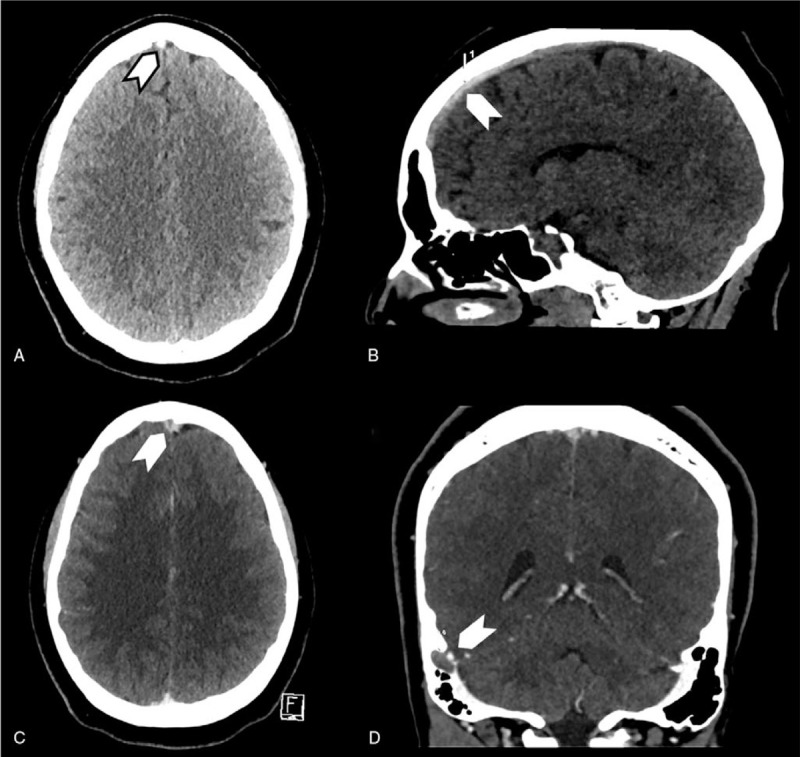
Patient (case 2) cerebral CT scan showing cerebral venous thrombosis. A (axial view) and B (sagittal view) showing spontaneous density of the anterior part of the superior sagittal sinus (arrows), C (axial view) and D (sagittal view) with contrast showing filling defect in the superior sagittal sinus with classical delta sign (C) and in the right sigmoid sinus (D) (arrows).

A 19-year-old woman with medical history of migraine, and morbid obesity (body mass index 39.4) was admitted on April 23, 2020 in Strasbourg University Hospital (Great East, France) for first tonic-clonic seizure. She did not use oral contraception, and had no family history of thrombosis. She complained of unusual headache, cough, and extreme asthenia for a week. Physical examination revealed only headache, without fever. Chest CT scan showed bilateral ground glass opacities affecting pulmonary parenchyma, evocative of COVID-19 (Fig. 3). SARS-Cov-2 infection was confirmed by reverse transcription PCR on a nasopharyngeal swab. Brain CT scan with venous angiography showed thrombosis of the superior sagittal sinus, frontal cortical veins, and right sigmoid sinus with no parenchymal consequences (Fig. 4). Cerebrospinal fluid analysis was normal. Blood tests showed only a moderate anemia (hemoglobin level of 10.6 g/dl), due to iron deficiency (ferritin level of 24 μg/L), and folate deficiency (level of 3.4 μg/L), with normal homocysteine level. Platelet count was at 425 G/L, leukocyte count at 6.5 G/L, with lymphocyte count at 3 G/L. C-reactive protein level was slightly increased at 11.5 mg/L. Coagulation tests showed decreased protein S activity (40%), and high D-Dimer level (2150 G/L), with normal PT, APTT, protein C, and antithrombin levels, no activated protein C resistance, and no antiphospholipid antibodies including antiβ2GP1 antibodies. Patient was treated, after admission in stroke unit, with anticoagulation therapy [low molecular weight heparin (LMWH) followed by vitamin K antagonist (VKA)], and antiepileptic drug (levetiracetam). Outcome was favorable at discharge with only minor persistent headache.

## 5 Discussion

While thrombosis is now known as a major feature of SARS-Cov-2 infection, report of CVT in the context of COVID-19 pandemia remains poor. Stroke is reported for only 2.5% of COVID-19 patients,^[3]^ contrasting with high incidence of VTE at least 10 times higher.^[2]^ CVT represents uncommon and particular location of VTE. In general population, annual CVT incidence is less than 2 per 100,000 persons.^[24]^ Studies assessing VTE incidence in COVID-19 included mainly patients admitted in ICU. Most of them were in a sedated condition, leading to ignore neurological signs and underestimate incidence of cerebrovascular diseases. Results from systematic cerebral imaging in these COVID-19 patients would be of high interest. More, we lack data about VTE incidence in patients with mild and moderate COVID-19, most being at home. Both reports describe patients admitted with intracranial hypertension headache and focal neurological deficit, allowing CVT diagnosis as early as admission. This emphasizes the causality link between thrombosis and COVID-19, excluding other pro thrombotic factors found in ICU as immobilization.

Thrombophilia is reported as the first cause of CVT in ISCVT study, concerning one third of patients.^[25]^ Our first patient presented slight decrease of antithrombin level, and deficit of protein S activity for the second one. However, these findings have to be interpreted with caution. Transient decrease of natural anticoagulants due to consumption is usual at the acute phase of thrombosis. Levels of these factors have to be checked a long time after treatment. Both patients presented other pro thrombotic factors as antiestrogen therapy or morbid obesity. But, simultaneous SARS-Cov-2 infection, with high pro thrombotic propensity, highly suggests COVID-19 played important roles in CVT development. Infections causing CVT became less frequent since antibiotic therapy. Most were head and neck infections, causing CVT through adjacency mechanisms. CVT, in our COVID-19 patients, would more reflect a general coagulation disorder. In view of typical pneumonia with ground glass opacities on chest CT scan, negative nasopharynx PCR in the first case was not at issue for diagnosis of SARS-CoV-2 infection.^[7,12,14,16,18,23,26]^ Interestingly enough, seriousness of CVT seems proportional to lung involvement in our both cases. CVT, and TVE, would not be only a complication of SARS-CoV-2 infection, but rather integral part and marker for COVID-19 seriousness.

**Figure 5:**
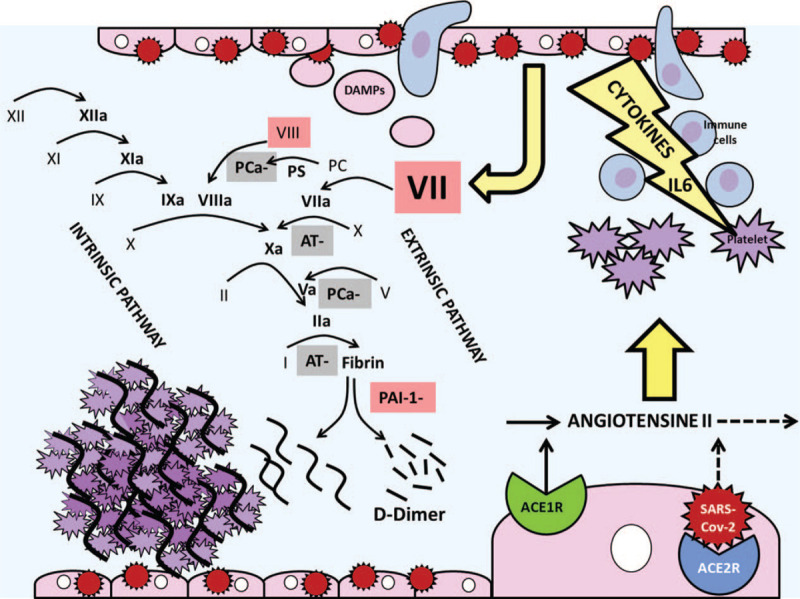
Pathogeny of thrombosis in SARS-Cov-2 infection. Binding of SARS-Cov-2 with the angiotensin converting enzyme 2 (ACE2) receptor on endothelium surface induces 2 pathways: (A) Activation of endothelium infiltrated by leukocytes and damage-associated molecular patterns (DAMPs) leads to immunothrombosis with cytokine storm, leukocyte and platelet recruitment, and activation of coagulation extrinsic pathway followed by intrinsic pathway^[28]^; (B) A rise of the angiotensin II level, pro inflammatory hormone, caused by a decrease of conversion, and increased synthesis due to ACE1 receptor shedding.^[31]^ The final goal is overproduction of fibrin and thrombosis, physiologic fibrinolysis leading to D-Dimer production. -: inhibition, a = activated, ACE 1,2 R = angiotensin converting enzyme 1 and 2 receptors, AT = antithrombin, DAMPs = damage-associated molecular patterns, PAI-1 = plasminogen activator inhibitor-1, PC = protein C, PS = protein S, coagulation factors underlined in red are activated, those underlined in grey are inhibited.

Thrombosis is implicated in SARS of COVID-19, the most classical complication requiring patient admission in ICU. Overproduced fibrin settles in lung alveoli preventing gas exchanges, and contributes to microvascular thrombosis accountable of pulmonary shunt.^[27,28]^ More, COVID-19 is a general thrombotic disease with microvascular thrombosis leading to multiple organ dysfunction syndrome, and macrovascular thrombosis.^[28]^ Thrombosis and SARS-CoV-2 are so linked as D-Dimer level is associated with mortality in COVID-19 patients.^[29]^ Infection of cells by SARS-CoV-2 is mediated by binding with the angiotensin converting enzyme (ACE) 2 receptor found in airway and lung epithelia, but also on overall endothelium.^[30,31]^ Interaction of SARS-Cov-2 with surface of endothelial cells is the key for initiation of immunothrombosis.^[28]^ Major consequences are inflammation and coagulation, inextricably linked in the thrombosis process during COVID-19.^[32]^ Inflammation is initiated by “cytokine storm” (IL-2, IL-6, IL-7, IL-10, G-CSF, IP-10, MCP-1, MIP-1A, and TNF-α) with recruitment of macrophages and monocytes.^[33]^ Inflammation is maintained due to endothelium infiltration by virus, immune cells, and production of damage-associated molecular patterns (DAMPs).^[31]^ Endothelium damage represents the first step of coagulation with activation of the extrinsic pathway followed by the intrinsic one, which final goal is fibrin production.^[28]^ Imbalance between pro thrombotic factors (including factor VII, VIII, von Willebrand factor, and presence of antiphospholipid antibodies),^[31,34,35]^ reduction of natural anticoagulant (antithrombin, protein C), and deficiency of fibrinolytic system (increased plasminogen activator inhibitor-1 (PAI-1) activity) leads to coagulation disorders referred under the term of “COVID-19 associated coagulopathy.”^[36]^ Moderate extension of PT and APTT was observed in about 20% of COVID-19 patients, and in particular in those who died.^[29]^ Thrombocytopenia, high D-Dimer, and fibrinogen levels are also observed, this latter being correlated with IL-6 level.^[36,37]^ Infection worsening leads subsequently to sepsis-induced coagulopathy and disseminated intravascular coagulation.^[38]^ These different stages of coagulopathy in COVID-19 promote much more thrombosis than hemorrhage.^[36]^ D-Dimer levels in COVID-19 would represent both inflammatory and thrombotic processes, this conferring its high prognostic significance.^[29]^ However, cytokine levels seem lower in COVID-19 than in other SARS. Other pathway involving angiotensin II, hormone with pro inflammatory properties, is suggested.^[31]^ An increase of angiotensin II levels is induced by ACE2 receptor binding with SARS-CoV-2 preventing the angiotensin II conversion, and leads to shedding of ACE1 receptors responsible for its synthesis. Rise of angiotensin II levels enhances immunothrombosis with cytokine release, leukocyte and platelet recruitment, and endothelium activation leading to factor VII and PAI-1 release with positive feedback loop.^[28]^ Immunothrombosis and the angiotensin II pathway leading to thrombosis in COVID-19 are summarized in the Figure 5.

The key for CVT treatment is anticoagulation therapy, with a preference for LMWH.^[39]^ LMWH allows achieving effective anticoagulation without delay.^[40]^ Anticoagulation therapy must not be discontinued or decreased in case of cerebral hemorrhage related to CVT. Indeed, hemorrhagic transformation of venous infarction is a sign of thrombosis seriousness. Decompressive craniectomy is highly recommended for patients with impending risk of cerebral herniation.^[39]^ Despite poor outcome described for our first patient, it should be kept in mind infarction due to CVT includes a major part of reversible edema, allowing good prognosis even if initial presentation could be serious.^[41]^ Secondary prevention for CVT is anticoagulation continued during a period for 3 and 12 months. VKA remains the preferred therapy.^[42]^ Recent study showed safety for dabigatran, but sample size was too small to demonstrate superiority or noninferiority.^[43,19]^ Prevention of VTE for patients with COVID-19 is not well established.^[44]^ A significant reduction of mortality has been demonstrated for patients with more severe SARS-Cov-2 infection treated with unfractionated heparin or LMWH, and interestingly, in particular for those with high D-Dimer levels.^[45]^ A combined anticoagulant and anti-inflammatory effect of heparin is suggested.^[33]^ Preventive anticoagulation therapy for patients admitted with severe and critical COVID-19 is recommended,^[46]^ but heparin daily dose remains under discussion. Anyway, basic coagulation parameters (platelet, D-dimer, fibrinogen levels, and PT) should be monitored in patients with severe COVID-19.^[37]^ Even less is known for VTE prevention in mild and moderate COVID-19 patients treated at home. Only hydration and mobilization are recommended in these cases.^[37]^ Involvement of fibrin production in development of severe SARS leaded some physicians to treat some COVID-19 patients with recombinant tissue plasminogen activator, but with only transient clinical improvement.^[47]^ For the moment no interaction has been observed between investigational drugs to treat COVID-19 and parenteral anticoagulation, but particular attention should be paid for VKA and direct oral anticoagulants.^[48]^

Prognosis for patients presenting CVT during COVID-19 seems especially poor.^[12–14,16,17,20,22]^ This could be explained by the high rate of mortality of patients with severe COVID-19 requiring ICU. But, most of these patients presented CVT involving deep venous system,^[12,13,18,20,22,23]^ known as being related to poor prognosis.^[25]^ Considerable time to diagnose CVT, and then to implement anticoagulation therapy may be incriminated. Indeed, headache is often encountered during COVID-19 leading physicians to omit this key symptom of CVT.^[49]^ Therefore, it seems crucial to better understand pathogeny and clinical course of CVT during COVID-19 to recommend strategy of diagnosis and treatment.

## 6 Conclusion

CVT, a life-threatening condition, can be considered as a major complication of COVID-19, particularly in view of the propensity of SARS-Cov-2 to cause thrombosis. CVT has to be searched in all patients presenting intracranial hypertension signs, whatever level of COVID-19 seriousness. Early diagnosis is all the more important as outcome of patients with CVT is related to implementation of anticoagulant therapy. Data are lacking about prevention of such serious thrombosis, in particular in patients with mild and moderate COVID-19.

## Author contributions

**Conceptualization:** Cécile Guendouz, Sébastien Richard.

**Data curation:** Cécile Guendouz, Véronique Quenardelle, Nolwenn Riou-Comte, Pascal Welfringer, Valérie Wolff, Lavinia Jager, Lisa Humbertjean Selton, Gioia Mione, Raoul Pop, Benjamin Gory, Sébastien Richard.

**Investigation:** Sébastien Richard.

**Validation:** Cécile Guendouz, Véronique Quenardelle, Nolwenn Riou-Comte, Pascal Welfringer, Valérie Wolff, Stéphane Zuily, Lavinia Jager, Lisa Humbertjean Selton, Gioia Mione, Raoul Pop, Benjamin Gory, Sébastien Richard.

**Visualization:** Sébastien Richard.

**Writing – original draft:** Cécile Guendouz, Véronique Quenardelle, Pascal Welfringer, Valérie Wolff, Stéphane Zuily, Lavinia Jager, Benjamin Gory, Sébastien Richard.
